# ^1^H NMR spectroscopy-based metabolomics analysis for the diagnosis of symptomatic *E. coli*-associated urinary tract infection (UTI)

**DOI:** 10.1186/s12866-017-1108-1

**Published:** 2017-09-21

**Authors:** Milena Lussu, Tania Camboni, Cristina Piras, Corrado Serra, Francesco Del Carratore, Julian Griffin, Luigi Atzori, Aldo Manzin

**Affiliations:** 1 Department of Biomedical Sciences, Microbiology and Virology Unit, University of Cagliari, S.S. 554, Bivio per Sestu, I-09042 Monserrato, Cagliari Italy; 2 Department of Medical Sciences and Public Health, University of Cagliari, Cagliari, Italy; 3 Faculty of Life Sciences, University of Manchester, Manchester, UK; 4 Department of Biochemistry, University of Cambridge, Cambridge, UK

**Keywords:** ^1^H NMR spectroscopy, UTI, *E. coli*, Acetate, Trimethylamine, ROC curve

## Abstract

**Background:**

Urinary tract infection (UTI) is one of the most common diagnoses in girls and women, and to a lesser extent in boys and men younger than 50 years. *Escherichia coli*, followed by *Klebsiella spp.* and *Proteus spp.*, cause 75-90% of all infections. Infection of the urinary tract is identified by growth of a significant number of a single species in the urine, in the presence of symptoms. Urinary culture is an accurate diagnostic method but takes several hours or days to be carried out. Metabolomics analysis aims to identify biomarkers that are capable of speeding up diagnosis.

**Methods:**

Urine samples from 51 patients with a prior diagnosis of *Escherichia coli*-associated UTI, from 21 patients with UTI caused by other pathogens (bacteria and fungi), and from 61 healthy controls were analyzed. The ^1^H-NMR spectra were acquired and processed. Multivariate statistical models were applied and their performance was validated using permutation test and ROC curve.

**Results:**

Orthogonal Partial Least Squares-discriminant Analysis (OPLS-DA) showed good separation (R^2^Y = 0.76, Q2=0.45, *p* < 0.001) between UTI caused by *Escherichia coli* and healthy controls. Acetate and trimethylamine were identified as discriminant metabolites. The concentrations of both metabolites were calculated and used to build the ROC curves. The discriminant metabolites identified were also evaluated in urine samples from patients with other pathogens infections to test their specificity.

**Conclusions:**

Acetate and trimethylamine were identified as optimal candidates for biomarkers for UTI diagnosis. The conclusions support the possibility of a fast diagnostic test for *Escherichia coli*-associated UTI using acetate and trimethylamine concentrations.

**Electronic supplementary material:**

The online version of this article (10.1186/s12866-017-1108-1) contains supplementary material, which is available to authorized users.

## Background

Uncomplicated urinary tract infection (UTI) is one of the most common diagnoses especially in young women who are sexually active, and although less frequent still reasonably common in older women, pregnant women and men [1, 2]. Uncomplicated cystitis and pyelonephritis are mainly caused by *Escherichia coli* (75% -95%) and possibly by other species of Enterobacteriaceae (such as *Proteus mirabilis* and *Klebsiella pneumoniae*) and *Staphylococcus saprophyticus* [3]. Other gram-negative and gram-positive species are rarely isolated in uncomplicated UTIs. Signs and symptoms of acute uncomplicated UTI include dysuria, urinary frequency or urinary urgency, loin pain, and eventually hematuria. The occurrence of other symptoms, such as vaginitis and urethritis suggests alternative diagnoses. The diagnosis of UTI is generally a clinical diagnosis based on symptoms and signs, and urine culture is usually not required to manage uncomplicated infections [1]. In fact, recent expert guidelines [4] state that for women without a history of a laboratory-confirmed UTI, a visit in a clinic, or ambulatory care facility for urinalysis or dipstick testing is appropriate. However, it is recommended that in symptomatic patients a negative dipstick result should be confirmed by a urine culture, urinalysis, or both [1, 4]. Urine culture is the gold standard for the microbiological diagnosis of UTI, and it is routinely used in most clinical laboratories [5]. As well, the method is labour intensive and time-consuming, and the turnaround time to obtain culture results is often exceeding the time requested to start the antimicrobial treatment. Moreover, only half of the symptomatic women have a UTI if the definition of infection is more than 10^5^ colony-forming units (CFU)/mL. Nevertheless, both urinalysis and culture are not able to predict clinical outcome in most women with clinical signs of infection, and recent evidence-based guidelines suggest that treating adult symptomatic women without laboratory testing does not increase adverse outcomes [6, 7]. Accordingly, given the cost and time constrains routine urine culture should be avoided to manage most uncomplicated UTI. Finally, prescriptions for UTI treatment have a significant impact on total antibiotic consumption and are associated both with an increase in expense for healthcare and with the spread of antibiotic resistance. Clearly, better and rapid diagnosis of UTI might prevent patients from being unnecessarily treated [4]. In the light of these recommendations, there is a need to develop a simpler, faster and cheaper tool to predict the causative organisms for bacterial UTI, or at least those suspected as the most likely source of UTI. For these reasons, any improvement in the diagnostic process of UTI would greatly impact future healthcare.

Recently, the availability of new technologies such as identification by matrix-assisted laser desorption/ionization time-of-flight mass spectrometry (MALDI-TOF) and the development of automated solutions designed for microbiology have had a positive impact on patient management and hospital costs, increasing productivity and quality, and reducing turnaround time and laboratory costs [8, 9]. Furthermore, application of high-resolution nuclear magnetic resonance spectroscopy (NMR) and mass spectrometry (MS), ultra-performance liquid chromatography, coupled with analytical and bioinformatics techniques, has enabled the metabolomics profiles of a number of diseases to be comprehensively investigated [10]. In particular, in the recent years ^1^H-NMR spectrometry has been used in the diagnosis of bacterial UTI, reaching an accuracy of nearly 99.5% using a multivariable prediction model. With this approach, a panel of biomarkers could act as indicators of UTI, thus providing a feasible method for diagnosis [11–16]. Here we confirm that NMR-based urinalysis is a useful and rapid method for the etiological diagnosis of *E. coli*-associated UTI that could be properly translated for clinical practice.

## Results

Based on the urine culture results, 51 patients with *E. coli*-associated UTI (*E. coli*-pos), 21 patients with non-*E. coli* UTI, and 61 healthy controls (CTRLs) were selected for the metabolomics analysis. Clinical and demographic characteristics of the patients and the healthy subjects are summarized in Table 1. Amongst the 72 patients positive for microbial infection, only 72% were positive for nitrite after dipstick urinalysis. On the other hand, about 93% resulted positive for the nonspecific inflammation marker leukocyte esterase. All urine samples underwent NMR analysis. A representative ^1^H NMR spectrum of urine obtained from *E. coli*-pos sample is reported in Additional file 1: Figure S1.

**Table 1:** Characteristics of the 133 subjects of the study

Characteristics	UTI patients (*E.coli*)	UTI patients (other pathogens)	Controls
	*N* = 51	*N* = 21	*N* = 61
Age, median ± SD	60 ± 20	73 ± 13.98	52 ± 18
Male, n (%)	9 (17.6)	8 (38.1)	26 (42.6)
Female, n (%)	42 (82.4)	13 (61.9)	35 (57.4)
Smoking, n (%)	12 (23.5)	missing values	18 (29.5)
Co-morbidity, n (%)			
Diabetes mellitus	3 (5.9)	5 (23.8)	4 (6.5)
BPCO	2 (3.9)	0 (−)	0 (−)
Malignancy	1 (1.9)	2 (9.5)	1 (1.6)
Arthritis	4 (7.8)	0 (−)	3 (4.9)
Hearth failure	0 (−)	1 (4.8)	0 (−)
Immunodeficiency	0 (−)	1 (4.8)	0 (−)
Urinary dipstick results			
Nitrite	37 (72.5)	13 (61.9)	1 (1.6)
Leukocyte esterase	49 (96.0)	18 (85.7)	3 (4.9)

In order to perform the multivariate statistical analysis, the data from *E. coli*-pos and CTRLs samples were split into training set and validation set. Training set was composed of 72 samples (31 *E. coli-*pos + 41 CTRLs) and the validation set of 40 samples. The multivariate statistical analysis was carried out using binned bucketed data.

**Fig. 1:**
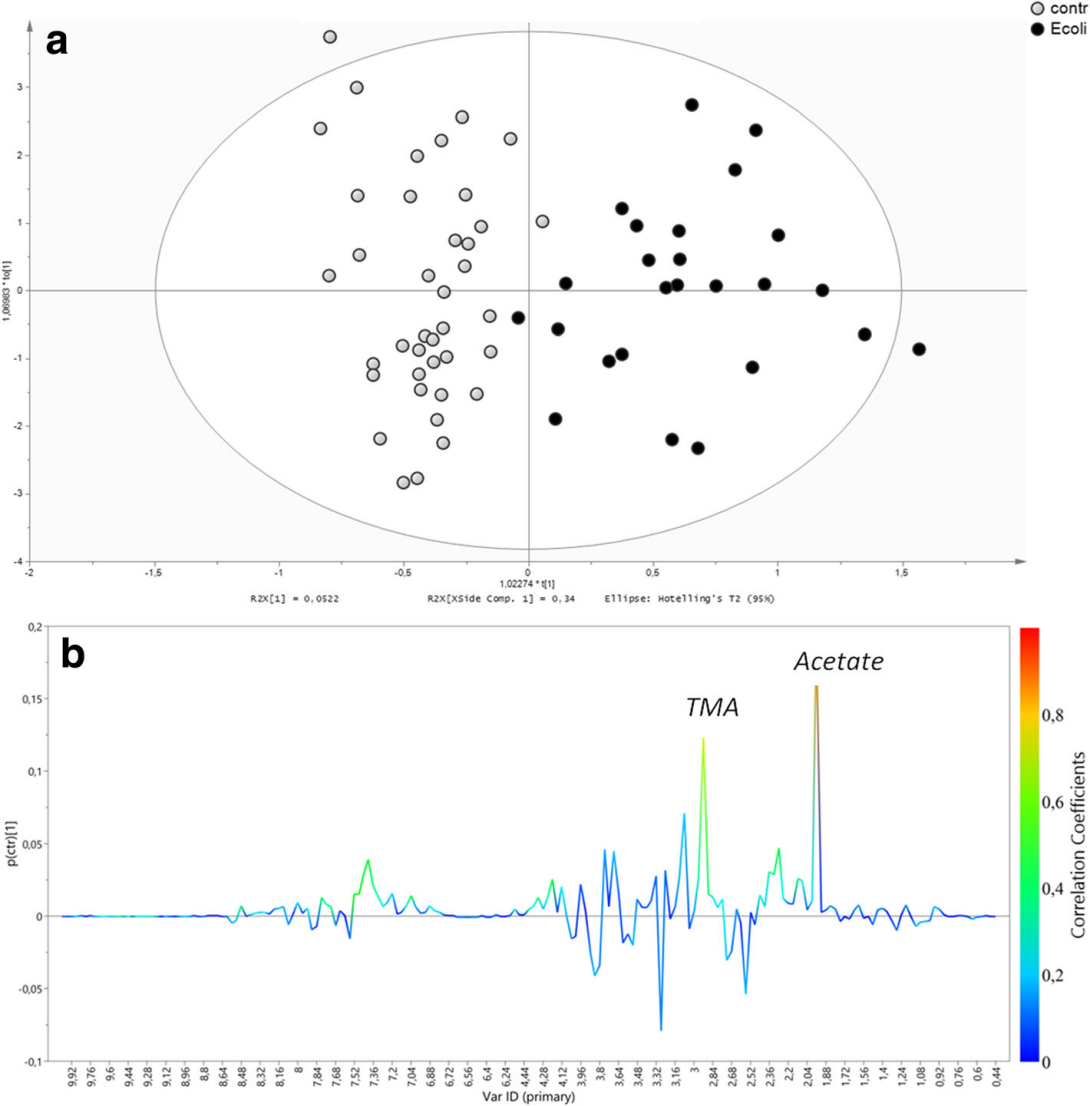
**a**) Distribution of ***E. coli***-associated UTI and healthy controls obtained with a OPLS-DA model. Controls (open circle), ***E. coli***-associated UTI (full circle). **b**). Color-coded coefficient loadings plots between the Controls and *E. coli*-associated UTI

To generate an overview of the dataset variation, a Principal Components Analysis (PCA) was first performed based on the normalized NMR spectral data obtained from urine samples. In order to get a better discrimination between the groups, orthogonal partial least square-discriminant analysis (OPLS-DA) was applied in our study. The OPLS-DA showed a clear separation of samples into two distinct groups, indicating that the *E. coli*-pos and CTRLs samples had a significant different metabolic profile. Score plots using the first two PCs were used to present a 2D representation of variations among the spectra (Fig. 1a).

The OPLS-DA model was established with one-predictive and one-orthogonal components, and showed good values of R^2^Y (0.76) and Q2 (0.45) and a *p* value < 0.001. Then, in order to test the validity of the OPLS-DA model, a permutation test (200 times) on the corresponding PLS-DA model was performed by using the same number of components (Additional file 2: Figure S2).

**Fig. 2:**
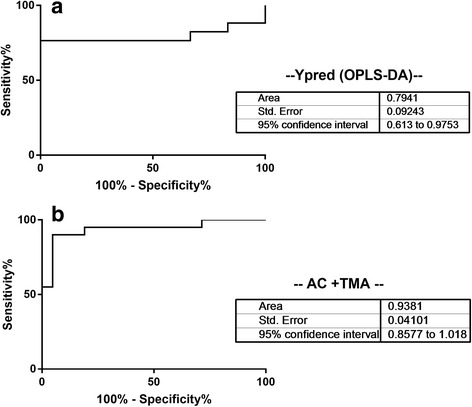
ROC Curve built using (**a**) Y predicted (OPSL-DA) and (**b**) Acetate and trimethylamine concentrations derived from patients with *E. coli*-associated UTI and healthy controls

The R2 and Q2 values derived from the permuted data were lower than the original values and the regression of Q2 line intersected at below zero, indicating the validation of the PLS-DA model (Q2 of - 0.131). By analyzing the coefficient loading plot color-based between the Controls and *E. coli*-associated UTI we selected regions of the spectra with a key role on the OPLS-DA (Fig. 1b). The validation set was then used. The classification of an external validation dataset using the model-parameters based on the training set provides information about the generalizability of the model. The predicted value of Y variable based on OPLS-DA was calculated for all prediction set and used as classifier. Receiver operating characteristic (ROC) curve analysis was applied to provide a measure of clinical utility. The area under the ROC curve (AUC) calculated using the Y-predicted value was 0.79 (95% CI 0.61-0.98) (Fig. 2a).

**Fig. 3:**
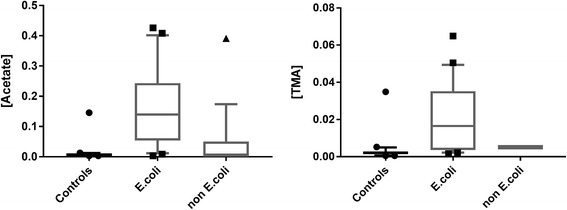
Box-and-whisker of relative concentration of controls, *E. coli*-associated UTI and non-*E. coli* UTI urine samples. Statistical significance was determined using the Mann-Whitney U test and a *p*-value <0.05 was considered statistically significant

Using the ChenomX software, the discriminant metabolites were identified and quantified. This method compares the integral of a known reference signal (trimethylsilyl propanoic acid, TSP), with signals derived from a documented database of about 350 compounds in order to determine concentrations relative to the reference signal. The univariate statistics were calculated using the Mann Whitney U test, with each metabolite concentration normalized to the creatinine concentration. The discriminant metabolites, their chemical shifts and the *p*-values are listed in Table 2. Acetate, Ac, and trimethylamine, TMA, were shown to be the most discriminatory metabolites. Box plots of relative concentrations for both metabolites are shown in Fig. 3, indicating the diversity of individual metabolites among different groups. The ROC curve build using both metabolites gave an AUC of 0.938 (95% CI 0.86-1.02) (Fig. 2b).

**Table 2:** List of discriminant metabolites based on the discriminant analysis (OPLS-DA) of Controls vs *E. coli*-pos

Metabolite	Chemical Shift (ppm)	*p* value
Acetate	1.93	<0.0001
Trimethilamine	2.89	<0.0001
Trimethilamine N-Oxide	3.28	NS
Citrate	2.54, 2.66	NS
Hippurate	3.97, 7.84, 7.55, 7.64	NS
Glycine	3.55	NS

*P*-values, derived by the Mann Withney univariate analysis, were calculated using metabolites concentrations normalized to the creatinine concentration

The concentrations of these metabolites were also evaluated in urine samples from patients with infections with other pathogens to test the specificity of their detection. These metabolites were used as classifiers and the area under the ROC curve was evaluated. The ROC analysis results are shown in Table 3. The AUC from ROC curve build comparing *E. coli*-pos vs. CTRLs samples was 0.92 (95% CI 0.81-1.0) for acetate and 0.89 (95% CI 0.79-0.99) for TMA, and 0.94 (% CI 0.86-1.0) combining both metabolites, respectively. This indicates that ROC curve by single metabolite concentrations are not performing as well as using them together. The use of both acetate and trimethylamine concentrations shows almost 100% sensitivity and 100% specificity. The comparison between *E. coli*-pos samples and group “All” (CTRLs + non-*E. coli*) shows an AUC of 0.89 (95% CI 0.80-0.98) for Ac, 0.80 (95% CI 0.67-0.94) for TMA, and 0.89 (95% CI 0.80-0.99) using both, respectively. The results get worse, in terms of AUC, when comparing *E. coli*-pos and non-*E. coli* samples.

**Table 3:** Comparison of ROC curves

	Acetate			Trimethylamine			Ac + TMA		
	AUC	95% CI	*p* value	AUC	95% CI	*p* value	AUC	95% CI	*p* value
*E. coli-*pos vs CTRLs	0.92	0.81-1.0	<0.0001	0.89	0.79-0.99	<0.0001	0.94	0.86-1.0	<0.0001
*E. coli-*pos vs non-*E. coli*	0.86	0.74-0.99	<0.0001	0.70	0.50-0.9	<0.0001	0.84	0.70-0.98	0.0003
*E. coli-*pos vs All	0.89	0.80-0.98	<0.0001	0.80	0.67-0.94	0.0002	0.89	0.80-0.99	<0.0001

Urine samples positive for *E. coli* infections were compared to CTRLs, non-*E. coli* and All patients

The area under the curve (AUC), the 95% confidence interval (CI), and the *p* values are reported. The ROC curves were built using Acetate and Trimethylamine, individually, or combining both concentrations

## Discussion

Urinary tract infections are a common problem in adults that frequently need prescriptions of laboratory tests and use of antibiotics. On the other hand, the diagnosis of UTI is mainly a clinical diagnosis, and is primarily based on symptoms and signs and should not rely exclusively on laboratory tests that detect the presence of bacteria and/or white cells in urine, but diagnosis based only on symptoms risks overestimating real UTI [17]. Dipstick urinalysis provides some support with leukocyte esterase and nitrite information. They can be nonspecific since the leukocyte esterase is a marker of granulocytes and not sensitive as not all urinary pathogens produce nitrite, sensitivity of nitrite being between 37 and 59% [18]. In our study, in fact, the two parameters showed better performances, though they did not reach sufficient levels of analytical sensitivity, as expected.

Most human diseases have characteristic modifications in the metabolite profiles of fluids prior and during the development of clinical symptoms [19]. Thus, metabolomics offers a unique tool to investigate the complete set of small molecules derived from biochemical changes due to ongoing disease. The identification of these low-molecular-weight biomarkers will result in the early and more accurate diagnosis, care of disease and, as hopefully expected, in effective therapeutic treatment [20–22]. NMR-based urinalysis for the screening of UTI with high accuracy and reproducibility has previously been described. *E. coli*-associated UTI, the most common scenario for all UTI, has benefited from NMR analysis which provides a valuable presumptive diagnosis for clinician judgment. Bacterial metabolic end-products were invariably observed in contaminated urine samples to date [23], and a set of metabolites were investigated and proposed as marker for bacterial UTI: in most studies, acetate and TMA were indicated as ideal urine biomarker for bacterial UTI and *E. coli*-associated UTI, in that their presence can be attributed to the metabolic effect of bacterial contamination of urine in the presence of ongoing disease [14, 15]. In particular, it is known that TMA production is the result of integrated metabolism between the host primary metabolome and microbes that reduce trimethylamine N-oxide (TMAO) using the bacterial trimethylamine N-oxide reductase in the urinary bladder, thus making TMA as a specific marker for bacterial metabolic activity and a valuable marker of *E. coli*-associated UTI [14, 15]. The use of TMA as a biomarker, due to its origin, rules out a possible contamination as a result of positive test. In this study, using a metabolomics approach the urinary profile of UTI caused by the gram-negative *E. coli* could be distinguished from that of the healthy controls, and also from that caused by other non-*E. coli* pathogens. We focused our study on *E. coli*-positive patients in order to validate the metabolomics approach in a well defined group of UTI caused by the most frequently detected pathogen. Indeed, we have shown that the method could be used to discriminate the metabolomic profiles of UTI caused by other pathogens, and further investigation will aim to validate the specificity of the approach in different clinical contexts (especially in the intensive care units).

Acetate and trimethylamine concentrations appear optimal candidates as biomarkers for *E. coli*-associated UTI diagnosis in the clinical setting, and this can have application in the selection of antibiotic treatment of the patients.

In fact, the results suggest that NMR-based diagnosis is a rapid, simple and safe test for the diagnosis of UTI and enables the prescription of medication before the microbiological diagnosis of UTI is confirmed. This advancement in clinical utility of non-cultured based diagnosis should be regarded as a paradigm shift in clinical medicine and in microbiological diagnosis.

## Conclusions

The study further confirms the value of NMR spectrometry as a diagnostic tool for *E. coli*-associated UTI. The method is validated for clinical purposes, easy to use, delivers reliable results with a rapid turn-around time, and has a high sensitivity and specificity compared to routine tests as urinalysis or dipstick testing. The discriminative model enabled acetate and trimethylamine to correctly provide information on bacterial contamination with *E. coli* with a high predictive ability. Clinical application of NMR spectroscopy has been previously assessed, and its role as in vitro diagnostic test highlighted in a great variety of diseases, including infections. One of the goal is to restrict the use of empirical antibiotic treatments to patients with UTI and to curtail the overuse of drugs that increase the spread of antibiotic resistance and the public health costs. A rapid, sensitive and cheap biochemical test will in future well acceptable once it is standardized and a precise cut-off level defined for the identification and/or differentiation of specific UTI.

## Methods

### Patients

Urine samples, collected over the course of two years, 2013-2014, at Policlinico Universitario di Monserrato-Cagliari (Italy) from a total of 133 subjects were analyzed: 51 patients with *E. coli*-associated UTI (*E. coli*-pos), 21 patients with UTI caused by other pathogens (*Enterococcus spp. = 6; Staphylococcus spp. = 4; Proteus spp. = 3; Candida spp. = 8*), and 61 healthy controls (CTRLs). All samples had previously been collected for routine mandatory diagnostic analysis from patients with acute uncomplicated cystitis and manifesting symptoms of dysuria, urinary frequency, or urinary urgency, and in a few cases, hematuria. Urine samples with negative or low colony counts and without evidence of inflammatory disease were used as the control group (Table 1). The following parameters were registered for each patient: the collection date, age, sex, identification of the bacteria strain associated with UTI, and the results of the antimicrobial susceptibility test. Mid-stream urine samples were obtained before starting antimicrobial therapy and analyzed using standard microbiological methods. Samples were collected and immediately aliquoted for the analysis after the addition of sodium azide 0.1% (*w*/*v*) to stop bacteria growth.

### Microbiological assays

Samples for urine culture were analysed within one hour of sampling; otherwise, they were stored at 4 °C and processed until 24 h after collection. All samples were inoculated in CLED agar as well as MacConkey agar and Sabouraud dextrose agar (all from bioMérieux, Marcy-l’Étoile, France), and were incubated under aerobic conditions for 18-48 h at 35 °C. A positive culture was defined as having a number of yielded colonies ≥10^3^ CFU/ml when associated with clinically significant signs in symptomatic patients and in light of the patient’s immunological status. Non-UTI controls were samples with no microbial growth from subjects with a negative history of UTI. The identification and antimicrobial susceptibility of the isolated strains were performed using automatized Vitek2 (bioMérieux, Marcy-l’Étoile, France). The ATCC 25922 *E. coli* was used for quality control and susceptibility defined in accordance with EUCAST recommendations (http://www.eucast.org/clinical_breakpoints/).

After sample collection, fractions of urine samples were added with a solution of 0.1% sodium azide, then centrifuged for 10 min at 4 °C at 12000 rpm to remove whole cell debris and to avoid contaminants. The supernatant was used for subsequent NMR analysis.

### NMR spectroscopy analysis

For each sample 630 μl-aliquots were collected from the supernatant and processed as previously described [24]. Samples were then transferred into a 5 mm NMR tube for analysis. The ^1^H-NMR spectra were acquired using a Varian Unity Inova 500 MHz spectrometer (Agilent Technologies, Santa Clara, CA, USA). The acquisition conditions of the NMR spectra were the following: standard temperature of 27 °C, 1D NOESY sequence with a 90° pulse of 10.4 μs, acquisition time of 1.5 s and spectral width of 6000 Hz. Free induction decay (FID) was acquired 128 times to increase signal-to- noise ratio. FIDs were weighted by an exponential function with a 0.5-Hz line broadening factor prior to Fourier Transformation. Finally, acquired spectra were phase and baseline corrected (Version 7.1.2, Mestrelab Research S.L.).

### Statistical analysis

The ^1^H-NMR spectra were reduced to regions of 0.04 ppm in the region 0.5–9.5 ppm, excluding urea and residual water region. Total area for each spectum bin was normalized to a constant sum of 100 to minimize the effects of variable concentration among different samples [25].

The resulting dataset was then analyzed by using SIMCA-P+ (Version 13.0, Umetrics, Umeå, Sweden). Multivariate statistical models were validate as follows: one-third of the samples were removed from the total set and used as a validation set, and the rest of the samples were used for constructing the training set. The class of samples in the validation dataset was then predicted based on the model built from the training set.

PCA was performed to get an overview of similarities/differences between sample profiles and to detect possible outliers.

Additionally, PLS-DA was applied to maximize the separation between samples and to identify subsets (linear combinations) of metabolic features associated with a specific sample class. OPLS-DA was applied to achieve a better interpretation of PLS-DA models, as it removes systematic variations from the data by placing them in orthogonal components and maximizing class separation in the OPLS-DA component [26]. A permutation test was performed using 200 permutations to check overfitting of the PLS-DA model. Once the supervised model has been estimated from the training set, the model can be used to predict new observations. All observation of the prediction-set can be predicted using the supervised model calculating the predicted value of Y variable. The Y predicted value could be used to build a classifier.

Six metabolites were identified as discriminant for the separation between the *E.coli*-pos and CTRLs samples from the S-Plot line and, then, quantified using ChenomX NMR Suite 7.1 (Chenomx Inc., Canada) [27]. Univariate statistical summaries and tests were performed based on the creatinine-normalized concentrations. The univariate statistics were calculated using the Mann Withney U test to estimate the significance of group differences. *P*-values of less than 0.05 were considered statistically significant.

The discriminant metabolites were also quantified in a group of 21 urine samples with infections with other pathogens in order to test *E. coli* specificity. Each metabolite was used to build a classifier to discriminate between *E. coli*-pos versus CTRLs, *E. coli*-pos versus non-*E. coli* and *E. coli*-pos versus “ALL” (CTRLs + non-*E. coli*).

The performance of the classifiers was assessed using a ROC curve performed using GraphPad Prism version 7.00 (GraphPad Software, La Jolla California USA, http://www.graphpad.com). ROC curves are summarized in a single value, the area under the curve (AUC), that ranges from 0 to 1.0. [28].

## Additional files

Additional file 1: Figure S1. Representative 1H NMR spectra of *E.Coli*-pos urine sample. 1 TSP; 2 lactate; 3 alanine; 4 acetate; 5 citrate; 6 dimethylamine; 7 trimethylamine; 8 creatinine; 9 histidine; 10 choline; 11 TMA N-Oxide; 12 taurine; 13 glycine; 14 urea; 15 phenylalanine;16 hippurate; 17 formate. (TIFF 156 kb)

Additional file 2: Figure S2. Statistical validation by permutation analysis using 200 different model permutations. (TIFF 240 kb)

## Abbreviations

Ac: Acetate
CFU: Colony-forming units
CTRLs: Healthy controls
FID: Free induction decay
MS: Mass spectrometry
NMR: Nuclear magnetic resonance spectroscopy
OPLS-DA: Orthogonal partial least square-discriminant analysis
PCA: Principal components analysis
ROC: Receiver operating characteristic
TMA: Trimethylamine
TSP: Trimethylsilyl propanoic acid
UTI: Urinary tract infection

## References

[1] Mehenert-Kay SA (2005). Diagnosis and management of uncomplicated urinary tract infections. Am Fam Physician.

[2] Wilson ML, Gaido L (2004). Laboratory diagnosis of urinary tract infections in adult patients. Clin Infect Dis.

[3] Tandogdu Z, Wagenlehner FM (2016). Global epidemiology of urinary tract infections. Curr Opin Infect Dis.

[4] Gupta K, Hooton TM, Naber KG, Wullt B, Colgan R, Miller LG, Moran GJ, Nicolle LE, Raz R, Schaeffer AJ, Soper DE; Infectious Diseases Society of America; European Society for Microbiology and Infectious Diseases. International clinical practice guidelines for the treatment of acute uncomplicated cystitis and pyelonephritis in women: A 2010 update by the Infectious Diseases Society of America and the European Society for Microbiology and Infectious Diseases. Clin Infect Dis. 2011;52:e103-e120. 10.1093/cid/ciq257 21292654

[5] Knottnerus BJ, Bindels PJ, Geerlings SE, Moll van Charante EP, ter Riet G (2008). Optimizing the diagnostic work-up of acute uncomplicated urinary tract infections. BMC Fam Pract.

[6] Lugtenberg M, Burgers JS, Zegers-van Schaick JM, Westert GP (2010). Guidelines on uncomplicated urinary tract infections are difficult to follow: perceived barriers and suggested interventions. BMC Fam Pract.

[7] Kim M, Lloyd A, Condren M, Miller MJ (2015). Beyond antibiotic selection: concordance with the IDSA guidelines for uncomplicated urinary tract infections. Infection.

[8] DeMarco ML, Ford BA (2013). Beyond identification: emerging and future uses for MALDI-TOF mass spectrometry in the clinical microbiology laboratory. Clin Lab Med.

[9] Croxatto A, Prod'hom G, Faverjon F, Rochais Y, Greub G (2016). Laboratory automation in clinical bacteriology: what system to choose?. Clin Microbiol Infect.

[10] Zhang A, Sun H, Yan G, Wang P, Wang X. Metabolomics for biomarker discovery: moving to the clinic. Biomed Res Int. 2015:354671. 10.1155/2015/354671 PMC4452245 26090402

[11] Gupta A, Dwivedi M, Nagana Gowda GA, Ayyagari A, Mahdi AA, Bhandari M, Khetrapal CL (2005). (1)H NMR spectroscopy in the diagnosis of Pseudomonas Aeruginosa-induced urinary tract infection. NMR Biomed.

[12] Gupta A, Dwivedi M, Mahdi AA, Gowda GA, Khetrapal CL, Bhandari M (2009). 1H-nuclear magnetic resonance spectroscopy for identifying and quantifying common uropathogens: a metabolic approach to the urinary tract infection. BJU Int.

[13] Gupta A, Dwivedi M, Mahdi AA, Khetrapal CL, Bhandari M (2012). Broad identification of bacterial type in urinary tract infection using 1H NMR spectroscopy. J Proteome Res.

[14] Lam CW, Law CY, Cheung SK, Lee KC, Sze KH, Leung KF, Yuen KY, To KK (2014). NMR-based metabolomic urinalysis: a rapid screening test for urinary tract infection. Clin Chim Acta.

[15] Nevedomskaya E, Pacchiarotta T, Artemov A, Meissner A, van Nieuwkoop C, van Dissel JT, Mayboroda OA, Deelder AM (2012). 1H NMR-based metabolic profiling of urinary tract infection: combining multiple statistical models and clinical data. Metabolomics.

[16] Lam CW, Law CY, Sze KH, Kelvin, To KK (2015). Quantitative metabolomics of urine for rapid etiological diagnosis of urinary tract infection: evaluation of a microbial–mammalian co-metabolite as a diagnostic biomarker. Clin Chim Acta.

[17] Schmiemann G, Kniehl E, Gebhardt K, Matejczyk MM, Hummers-Pradier E (2010). The diagnosis of urinary tract infection: a systematic review. Dtsch Arztebl Int.

[18] Sultana RV, Zalstein S, Cameron P, Campbell D (2001). Dipstick urinalysis and the accuracy of the clinical diagnosis of urinary tract infection. J Emerg Med.

[19] Lenz EM, Bright J, Wilson ID, Morgan SR, Nash AFP (2003). A 1H NMR-based metabonomic study of urine and plasma samples obtained from healthy human subjects. J Pharm Biomed Anal.

[20] Tsutsui H, Maeda T, Min JZ, Inagaki S, Higashi T, Kagawa Y, Toyo'oka T (2011). Biomarker discovery in biological specimens (plasma, hair, liver and kidney) of diabetic mice based upon metabolite profiling using ultra-performance liquid chromatography with electrospray ionization time-offlight mass spectrometry. Clin Chim Acta.

[21] Zhang A, Sun H, Yan G, Wang P, Han Y, Wang X (2014). Metabolomics in diagnosis and biomarker discovery of colorectal cancer. Cancer Lett.

[22] Zhang A, Sun H, Wu X, Wang X (2012). Urine metabolomics. Clin Chim Acta.

[23] Zhang A, Sun H, Han Y, Yuan Y, Wang P, Song G, Yuan X, Zhang M, Xie N, Wang X (2012). Exploratory urinary metabolic biomarkers and pathways using UPLC-Q-TOF-HDMS coupled with pattern recognition approach. Analyst.

[24] Fanos V, Pintus MC, Lussu M, Atzori L, Noto A, Stronati M, Guimaraes H, Marcialis MA, Rocha G, Moretti C, Papoff P, Lacerenza S, Puddu S, Giuffrè M, Serraino F, Mussap M, Corsello G (2014). Urinary metabolomics of bronchopulmonary dysplasia (BPD): preliminary data at birth suggest it is a congenital disease. J Matern Fetal Neonatal Med.

[25] Ross A, Schlotterbeck G, Dieterle F, Senn H, Lindon JC, Nicholson JK, Holmes E (2007). NMR spectroscopy techniques for application to Metabonomics, *p* 55–112. The handbook of Metabonomics and Metabolomics.

[26] Trygg J, Wold S (2002). Orthogonal projections to latent structures (O-PLS). J Chemom.

[27] Weljie A, Newton MJ, Mercier P, Carlson E, Slupsky CM (2006). Targeted profiling: quantitative analysis of 1H NMR metabolomics data. Anal Chem.

[28] Berrar D, Flach P (2012). Caveats and pitfalls of ROC analysis in clinical microarray research (and how to avoid them). Brief Bioinform.

