# Supplementary figures and images for: 1H NMR spectroscopy-based metabolomics analysis for the diagnosis of symptomatic E. coli-associated urinary tract infection (UTI)
Source: BMC Microbiol. 2017 Sep 21;17:201. doi: 10.1186/s12866-017-1108-1 (PMC5609053; doi:10.1186/s12866-017-1108-1)

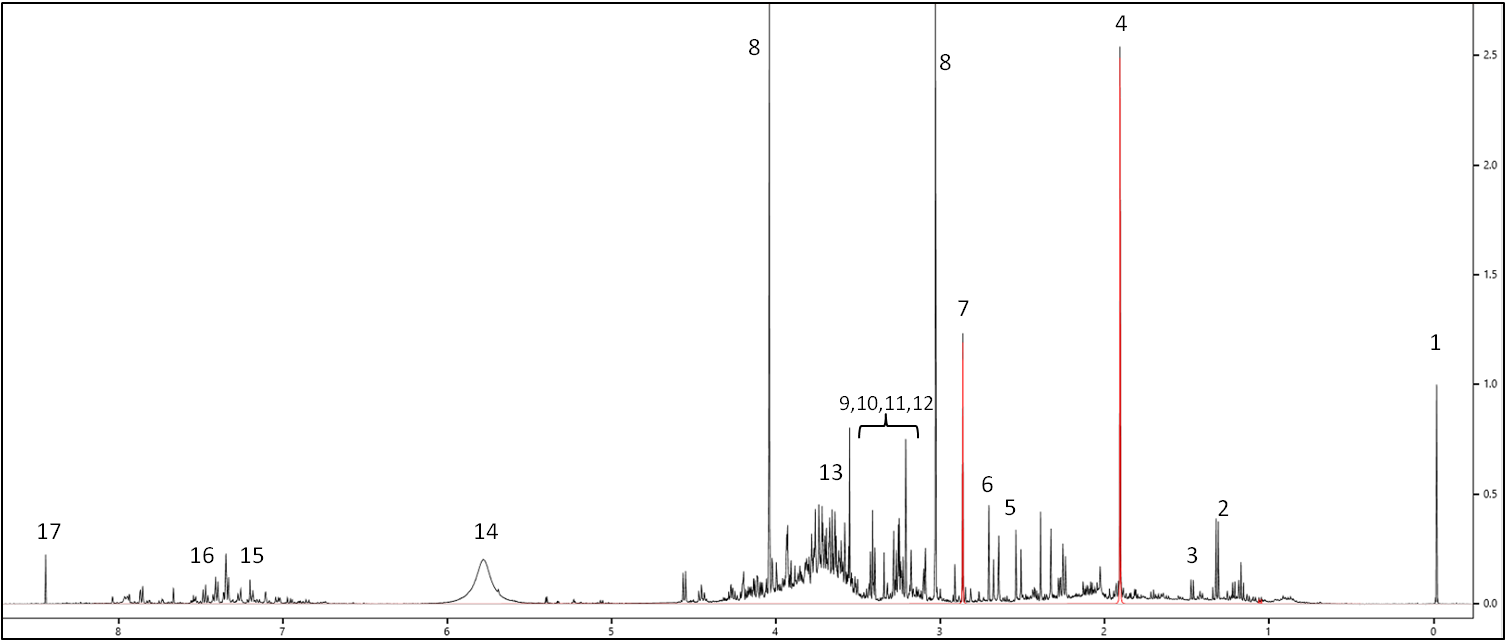

Supplement: Supplementary file 1 — Representative 1H NMR spectra of E.Coli-pos urine sample. 1 TSP; 2 lactate; 3 alanine; 4 acetate; 5 citrate; 6 dimethylamine; 7 trimethylamine; 8 creatinine; 9 histidine; 10 choline; 11 TMA N-Oxide; 12 taurine; 13 glycine; 14 urea; 15 phenylalanine;16 hippurate; 17 formate. (TIFF 156 kb) [file 12866_2017_1108_MOESM1_ESM.tif]

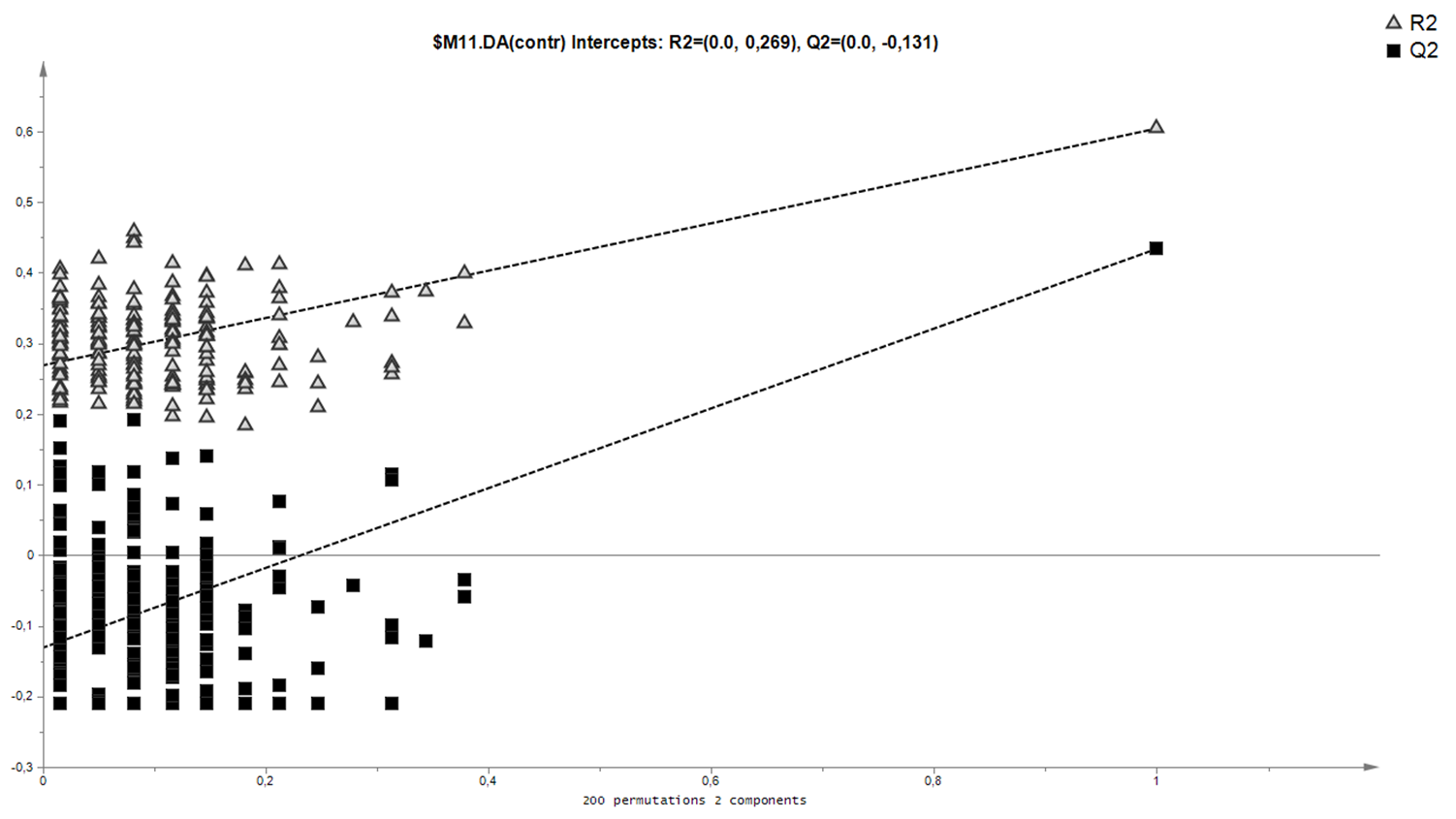

Supplement: Supplementary file 2 — Statistical validation by permutation analysis using 200 different model permutations. (TIFF 240 kb) [file 12866_2017_1108_MOESM2_ESM.tif]
